# Supplementary material for: Experimental In Vitro Microfluidic Calorimetric Chip Data towards the Early Detection of Infection on Implant Surfaces
Source: Sensors (Basel). 2024 Feb 5;24(3):1019. doi: 10.3390/s24031019 (PMC10857106; doi:10.3390/s24031019)
Supplement: Supplementary file 1 [file sensors-24-01019-s001.zip › sensors-2809354-supplementary.pdf]

Supplementary Information

Pictures of the thermally not-stabilized system and thermally stabilized system shown in Figure S1 and S2, both of the microfluidic chip and also of the experimental environment, as sketched in Figure 3 in the main text. The pictures in Figure S1 are taken in the temperature-controlled room where the whole room is set to 37°C. As shown in Figure S1 a) and c), the microfluidic chip is placed directly in the room without further thermal stabilization around the microfluidic chip.

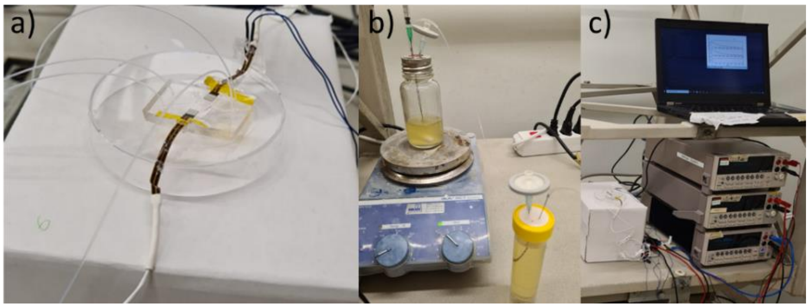

Figure S1. Thermally not-stabilized system. a) Picture of microfluidic chip in incubation room. b) Picture of the media containing bacteria and sterile media, as also shown in Figure 3 in the main text. c) Picture of the sensor readout system.

In contrast, the picture in Figure S2 b) shows the PMMA box surrounding the microfluidic chip and the placement inside an incubator set to 37°C.

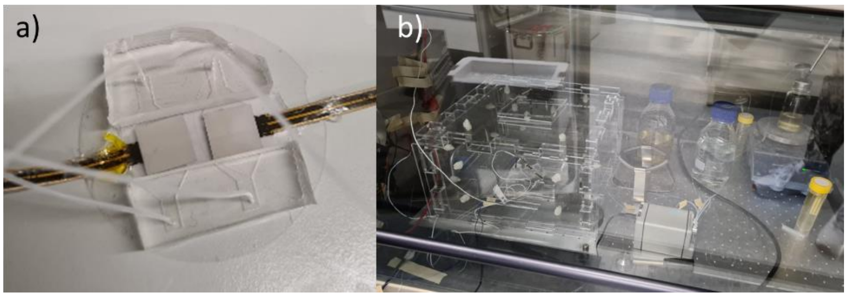

Figure S2. Thermally stabilized system [1]. a) Picture of the microfluidic chip. b) Picture of the incubator containing the PMMA box (in which the microfluidic chip is set), the different bottles and the peristaltic pump.

References

1. Vehusheia, S.L.K.; Roman, C.; Braissant, O; Arnoldini, M.; Hierold, C., Enabling direct microcalorimetric measurement of metabolic activity and exothermic reactions onto microfluidic platforms via heat flux sensor integration. Nature Microsystems and Nanoengineering 2023, 9, 56
